# Year-Long Cannabis Use for Medical Symptoms and Brain Activation During Cognitive Processes

**DOI:** 10.1001/jamanetworkopen.2024.34354

**Published:** 2024-09-18

**Authors:** Debbie C. L. Burdinski, Alisha Kodibagkar, Kevin Potter, Randi M. Schuster, A. Eden Evins, Satrajit S. Ghosh, Jodi M. Gilman

**Affiliations:** 1 McGovern Institute for Brain Research, MIT, Cambridge, Massachusetts; 2 Harvard Medical School, Boston, Massachusetts; 3 University of Pennsylvania School of Engineering and Applied Science, Philadelphia; 4 Center for Addiction Medicine, Massachusetts General Hospital, Boston; 5 Department of Otolaryngology–Head and Neck Surgery, Harvard Medical School, Boston, Massachusetts; 6 Athinoula A. Martinos Center for Biomedical Imaging, Department of Radiology, Massachusetts General Hospital, Boston

## Abstract

**Importance:**

Cannabis is increasingly being used to treat medical symptoms, but the effects on brain function in those using cannabis for these symptoms are not known.

**Objective:**

To test whether 1 year of cannabis use for medical symptoms after obtaining a medical cannabis card was associated with increased brain activation during working memory, reward, and inhibitory control tasks, areas of cognition affected by cannabis.

**Design, Setting, and Participants:**

This cohort study was conducted from July 2017 to July 2020 among participants from the greater Boston area who were recruited as part of a clinical trial of individuals seeking medical cannabis cards for anxiety, depression, pain, or insomnia symptoms. Participants were aged between 18 and 65 years. Exclusion criteria were daily cannabis use and cannabis use disorder at baseline. Data analysis was conducted from August 2021 to April 2024.

**Main Outcomes and Measures:**

Outcomes were whole brain functional activation during tasks involving working memory, reward, and inhibitory control at baseline and after 1 year of medical cannabis card ownership.

**Results:**

Imaging was collected from participants before and 1 year after obtaining medical cannabis cards, with 57 participants at baseline (38 female [66.7%]; 6 [10.5%] Black and 45 [78.9%] White participants; 1 [1.8%] Hispanic participant; median [IQR] age, 34.0 [24.0-51.0] years) and 54 participants at 1 year (37 female [68.5%]; 4 [7.4%] Black and 48 [88.9%] White participants; 1 [1.9%] Hispanic participant, median [IQR] age, 36.5 [25.0-51.0] years). Imaging was also collected in 32 healthy control participants at baseline (22 female [68.8%]; 2 [6.2%] Black and 27 [84.4%] White participants; 3 [9.4%] Hispanic participants; median [IQR] age, 33.0 [24.8-38.2] years). In all groups and at both time points, functional imaging revealed canonical activations of the probed cognitive processes. No statistically significant difference in brain activation between the 2 time points (baseline and 1 year) in those with medical cannabis cards and no associations between changes in cannabis use frequency and brain activation after 1 year were found.

**Conclusions and Relevance:**

In this cohort study of adults obtaining medical cannabis cards for medical symptoms, no significant association between brain activation in the areas of cognition of working memory, reward, and inhibitory control and 1 year of cannabis use was observed. The results warrant further studies that probe the association of cannabis at higher doses, with greater frequency, in younger age groups, and with larger, more diverse cohorts.

## Key Points

**Question:**

Is there an association between year-long cannabis use for medical symptoms and brain activation during cognitive processes implicated in cannabis use?

**Findings:**

In a cohort study of adults who newly obtained medical cannabis cards for symptoms of depression, anxiety, pain, or insomnia, functional magnetic resonance imaging measures during working memory, reward, and inhibitory control tasks did not differ statistically from baseline to 1 year and were not associated with changes in cannabis use frequency.

**Meaning:**

The absence of activation differences in this study suggests that adults using cannabis for medical symptoms over 1 year may not experience significant changes within reward, working memory, or inhibitory control domains.

## Teaser

This cohort study evaluates whether there is an association between 1 year of cannabis use for medical symptoms and brain activation during working memory, reward, and inhibitory control tasks.

## Introduction

Accumulating evidence has shown that regular cannabis use can alter brain function, especially in networks that support working memory, cognitive control, and reward processing.^1^ Several prior reviews have described the functional impact of chronic cannabis use in both adults and adolescents,^2,3,4^ largely concluding that the domains of executive functioning and memory are most strongly affected by regular cannabis use.^5,6^ However, most of the evidence for brain changes with cannabis use is derived from between-group brain differences between those who use cannabis and those who do not, rather than from longitudinal changes at pre– and post–cannabis use time points, raising the question of whether preexisting differences between those who use cannabis and those who do not underlie observed changes. Longitudinal studies, such as the Adolescent Brain Cognitive Development Study,^7^ are under way. To date, however, few studies are focused on adults using cannabis to treat medical symptoms. Little is known about the effects of cannabis on the brain in medical populations, who may also experience illness-related cognitive weaknesses and may have different use patterns and age ranges compared with recreational users.

Delta-9-tetrahydrocannabinol (THC), the main psychoactive compound in cannabis, binds to endogenous cannabinoid CB1 receptors located in brain regions such as the hippocampus, amygdala, basal ganglia, prefrontal cortex, substantia nigra, and globus pallidus,^8,9^ making frontal-limbic neurocircuitry particularly susceptible to cannabis-related effects in the brain.^10^ Specifically, THC binding inhibits the release of neurotransmitters usually modulated through endocannabinoids.^11^ Many factors can modulate THC’s impact on the brain, including duration, frequency and quantity of use, age of initiation, potency, accompanying cannabidiol content, presence of cannabis use disorder (CUD), concurrent use of other substances, and sex and genetics.^12^

The question of how cannabis affects the brain is particularly relevant to those using cannabis to treat medical symptoms. Currently in the United States, 38 states and the District of Columbia have medical cannabis programs, and enrollment in medical cannabis programs increased 4.5-fold from 2016 to 2020.^13^ In Massachusetts, obtaining a medical cannabis card (MCC) gives patients access to tax-exempt cannabis purchases and additional medical dispensaries. However, evidence for the effectiveness of plant-based cannabis for any medical condition is sparse.^14^ In dispensaries, a myriad of products (eg, candies, gummies, smoked, vaped) are available to those using medically, and the neural effects of these products are unknown.

We sought to describe cognitive and brain-based associations with cannabis use in a longitudinal sample of participants obtaining MCCs and beginning to use cannabis for symptoms of anxiety, depression, pain, and insomnia. We previously published a pragmatic randomized clinical trial (RCT) of MCCs for medical symptoms to assess their effect on target symptoms when compared with a waitlist control group.^15,16^ In the current cohort study, we describe a longitudinal analysis of task-based functional magnetic resonance imaging (fMRI) data from the arm of the clinical trial in which participants were assigned to obtain MCCs immediately. We explore the extent to which cannabis was associated with brain activation during cognitive processes previously implicated in cannabis use, using neuroimaging tasks that probe working memory, reward processing, and inhibitory control. We hypothesized, based on previous literature,^17,18,19^ that 1 year of cannabis use would be associated with generally increased activation in brain regions underlying these processes, and that an increase in cannabis use frequency would be associated with this increased activation, with few associated differences in task performance over the study period.

## Methods

### Study Recruitment

The participants of this observational cohort study were recruited for a pragmatic, single-site, single-blind, RCT assessing patients seeking MCCs in the greater Boston area from July 1, 2017, to July 31, 2020 (NCT03224468).^15,16^ Participants were between the ages of 18 and 65 years and were seeking to obtain MCCs for the first time for depression, anxiety, pain, or insomnia symptoms, the most commonly reported symptoms in those seeking cannabis for symptom management. Exclusion criteria included daily cannabis use, CUD diagnosis at screening or baseline, cancer, psychosis, and current substance use disorders (except for mild or moderate alcohol use disorder and nicotine use disorder).

### Study Protocol

Participants were randomized to either receive their MCC immediately or to delay acquisition by 12 weeks. Only the immediate MCC group received fMRI scans at baseline and at 1 year, and these data are presented here. Demographic variables were collected at baseline through self-report and included sex, age, race, ethnicity, years of education, the primary symptom for seeking MCCs, and handedness. Race and ethnicity were categorized as suggested by the National Institutes of Health for reporting and research purposes: for race, American Indian or Alaska Native, Black or African American, Asian, Native Hawaiian or Other Pacific Islander, and White; for ethnicity, Hispanic or Latino or not Hispanic or Latino. Due to limited numbers, Asian, Native Hawaiian or other Pacific Islander, and multiracial were collapsed into one category labeled other. Cannabis use metrics were collected at baseline and at 2, 4, 12, 24, and 52 weeks and included past-month frequency of cannabis use and a validated scale to assess CUD, the Cannabis Use Disorder Identification Test Revised (CUDIT-R). In addition, urinalysis for cannabis metabolites was conducted at the time of study visits. Structural and functional brain imaging data were collected in the MCC group (70 participants) (eFigure 1 in Supplement 1) at baseline and 1 year later. At baseline, imaging was also collected in an age- and sex-matched healthy control (HC) group (32 participants). fMRI tasks included a working memory task (N-back), a reward processing task (monetary incentive delay [MID]), and an inhibitory response task (stop signal task [SST]).^20,21,22^ The eMethods in Supplement 1 provide a description of the experimental paradigm (eFigures 2-4 in Supplement 1). Participants provided written informed consent and were financially compensated for their participation in the study. The clinical trial, as part of which data for this cohort study were collected, was approved by the Massachusetts General Brigham institutional review board. This report focuses on task-based functional imaging, which followed a pretest-posttest design with a control group at baseline. Clinical outcomes of the RCT are reported elsewhere.^15,16^ This study follows the Strengthening the Reporting of Observational Studies in Epidemiology (STROBE) reporting guidelines.

### Statistical Analysis

Demographic metrics were compared across 3 groups (HC participants, MCC participants imaged at both time points, and MCC participants who only received a scan at 1 time point) using a Kruskal-Wallis test for numerical variables, a Fisher exact test for categorical variables with less than 5 observations in a category, and a χ^2^ test for all other categorical variables. Cannabis metrics in the MCC participants who were imaged at both time points were compared across the 2 time points (baseline and 1 year) using a linear mixed-effects model with a participant-varying intercept to account for repeated measures and age and sex as covariates.

To analyze behavioral performance of tasks, differences between the HC and MCC groups at baseline and between the 2 time points of the MCC participants were assessed. For the N-back task, we analyzed reaction time and accuracy across the 2-back, 0-back, and combined stimuli using a linear regression model with age and sex as covariates. For the SST, we analyzed stop signal reaction time (SSRT; the inferred mean latency between the stop signal and response inhibition) via an additive multilevel linear model. Across-time models also included a participant-varying intercept to account for repeated measures. The MID task did not include a behavioral component, as response periods were dynamically updated throughout a run to maintain a similar accuracy across participants.

MRI data were preprocessed using version 23.0.1 of the fMRIPrep software, which included head-motion estimation, slice time correction, field map–based distortion correction, echo-planar imaging to T1 registration and resampling to both Montreal Neurological Institute volumetric and grayordinate space.^23,24^ The eMethods in Supplement 1 provide details on MRI acquisition and preprocessing.

Two general linear model analyses were conducted, one in volumetric and the other in grayordinate space, using the Python package Nilearn version 0.9.2.^25,26^ First-level linear regression modeling removed further noise and modeled typical task contrasts. Individual effect sizes for the contrasts were passed to a group-level linear regression model to assess group averages at a given time point, differences across groups and time points, and the role of changes in cannabis use frequency. Covariates included sex, age, and past-month cannabis use frequency, mean-centered for numerical variables. Whole brain activation was compared between MCC participants and a matched control group at baseline using a contrast between group-level intercepts. For the across-time analyses of the MCC group, observations were limited to those participants with imaging at both time points. We assessed whether, on average, there was a whole brain activation difference between baseline and 1 year in an individual, controlling for baseline cannabis use frequency to account for individual differences in use at the outset of the study. Furthermore, the association of the change in cannabis use frequency across time with whole brain activation at 1 year was assessed, adding the additional covariates of brain activation and cannabis use frequency at baseline. Of note, repeated measures in the across-time analysis were accounted for by including baseline values as covariates or by using a change score.

Quality control metrics derived from MRIQC (version 0.16.1) were used to exclude runs of lower quality prior to running group-level analyses, which led to varying sample sizes across tasks.^27^ The effect of quality control was assessed by running the analyses with varying exclusion criteria. Effect sizes at the group level were standardized for visualization purposes.^28,29^ The eMethods in Supplement 1 provide details on the general linear modeling approach and quality control metrics.

The statistical significance level was set to *P* < .05 for all analyses. Multiple comparisons were addressed by controlling the false-discovery rate (FDR) at .05 using the Benjamini-Hochberg procedure. Analyses were run in R version 4.3.3 (R Project for Statistical Computing) and Python version 3.9.13 (Python Software Foundation). Data analysis was conducted from August 2021 to April 2024.

## Results

### Participant Characteristics

Of 120 MCC participants in the parent trial, brain imaging data were collected in 70 as well as in 32 control participants (eFigure 1 in Supplement 1). A total of 35 MCC participants opted out of the MRI protocol, 11 were excluded due to having CUD at baseline, and 4 were lost to follow-up. At baseline, 57 MCC participants (38 female [66.7%]; 6 [10.5%] Black and 45 [78.9%] White participants; 1 [1.8%] Hispanic participant; median [IQR] age, 34.0 [24.0-51.0] years) and 32 control participants (22 female [68.8%]; 2 [6.2%] Black and 27 [84.4%] White participants; 3 [9.4%] Hispanic participants; median [IQR] age, 33.0 [24.8-38.2] years) were imaged. After 1 year, 54 MCC participants (37 female [68.5%]; 4 [7.4%] Black and 48 [88.9%] White participants; 1 [1.9%] Hispanic participant, median [IQR] age, 36.5 [25.0-51.0] years) were imaged. Of all MCC participants, 41 presented for imaging at both time points (28 female [68.3%]; 2 [4.9%] Black and 37 [90.2%] White participants; 1 [2.4%] Hispanic participant; median [IQR] age, 38.0 [26.0-51.0] years). MCC participants presenting for 1 scan, MCC participants presenting for 2 scans, and HC participants did not differ significantly in any of the characteristics assessed (Table). All cannabis use metrics, including CUDIT-R summed score, cannabis use frequency per month, and positive urine THC, were greater at 1 year than at baseline in the MCC group (eTable 1 in Supplement 1).

**Table.** Characteristics of the Study Participants

Characteristic	Participants, No. (%)			
	HC at Baseline (n = 32)^a^	MCC		
		Baseline (n = 57)^b^	1 y (n =54)^c^	Paired (n = 41)^d^
Sex				
Female	22 (68.8)	38 (66.7)	37 (68.5)	28 (68.3)
Male	10 (31.2)	19 (33.3)	17 (31.5)	13 (31.7)
Age, median (IQR)	33.0 (24.8-38.2)	34.0 (24.0-51.0)	36.5 (25.0-51.0)	38.0 (26.0-51.0)
Race				
Black	2 (6.2)	6 (10.5)	4 (7.4)	2 (4.9)
Other^e^	3 (9.4)	6 (10.5)	2 (3.7)	2 (4.9)
White	27 (84.4)	45 (78.9)	48 (88.9)	37 (90.2)
Ethnicity				
Hispanic or Latino	3 (9.4)	1 (1.8)	1 (1.9)	1 (2.4)
Education, median (IQR), y	17.5 (16.0-19.2)	16.0 (16.0-18.0)	16.5 (16.0-18.0)	17.0 (16.0-18.0)
Primary symptom				
Depression or anxiety symptoms	NA	27 (47.4)	24 (44.4)	19 (46.3)
Insomnia symptoms	NA	12 (21.1)	13 (24.1)	9 (22.0)
Pain symptoms	NA	18 (31.6)	17 (31.5)	13 (31.7)
Handedness				
Right-handed	29 (90.6)	49 (86.0)	46 (85.2)	35 (85.4)

Abbreviations: HC, healthy control; MCC, medical cannabis card; NA, not applicable.

^a^ HC baseline corresponds to the imaging control group at baseline.

^b^ MCC baseline corresponds to the MCC group’s participants imaged at baseline.

^c^ MCC 1 year corresponds to the MCC group’s participants imaged at 1 year.

^d^ MCC paired corresponds to the MCC group’s participants imaged at both time points.

^e^ Other includes individuals identifying as Asian, Native Hawaiian or other Pacific Islander, and multiracial.

### N-Back Task Behavioral and Imaging Results

At baseline, no performance differences in either accuracy or reaction time were observed between HC and MCC participants. MCC participants had a significantly faster mean (SD) 2-back reaction time at 1 year (540 [14] ms) compared with baseline (582 [13] ms) (β = −42.5; SE, 14.6; *P* = .04), and no difference in any of the other behavioral measures (eTable 2 in Supplement 1).

**Figure 1.**
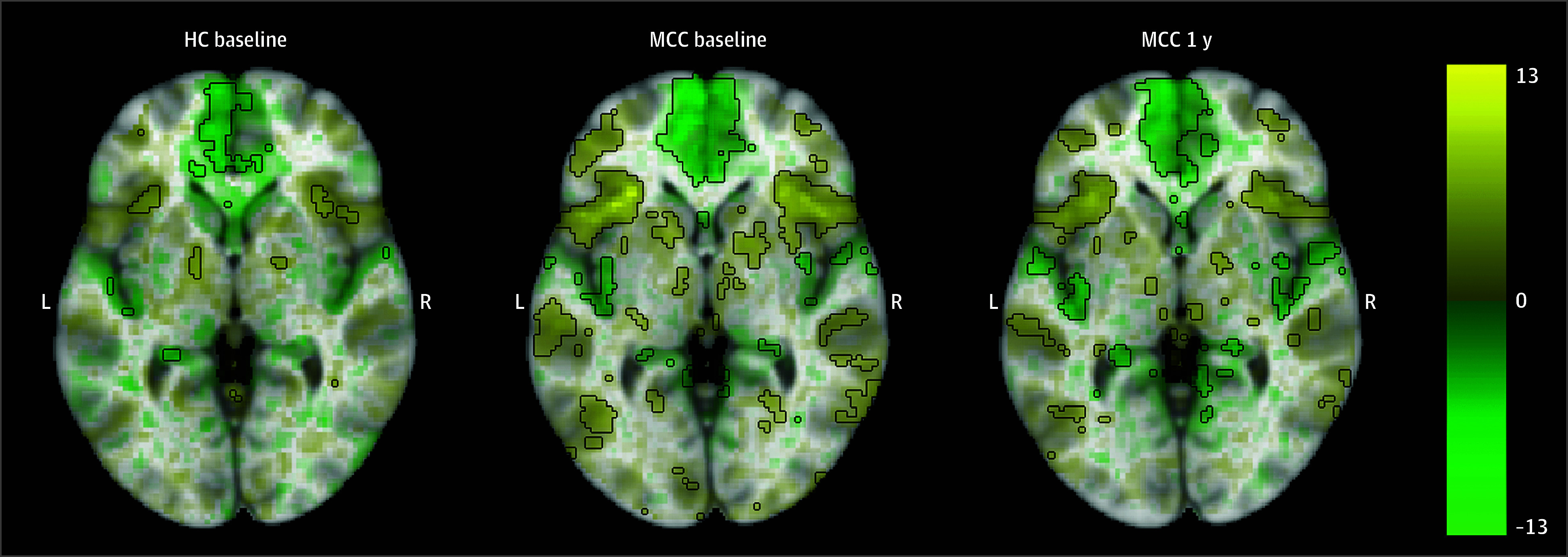
Brain Activation for the N-Back Task’s 2-Back vs 0-Back Contrast Across Groups and Time Points The healthy control (HC) group at baseline (22 participants), the medical cannabis card (MCC) group at baseline (40 participants), and the MCC group at 1 year (40 participants) did not show activation differences between the 2 groups at baseline or between the 2 time points of the MCC group. Cannabis use frequency changes were not associated with brain activation at 1 year. Voxel-wise average brain activation, colored by effect size and opacity-scaled by *z* scores with the significance threshold (false-discovery rate *P* < .05) outlined, for the 2-back vs 0-back contrast of the N-back task. The *z* thresholds were 3.05 for the HC group at baseline, 2.47 for the MCC group at baseline, and 2.60 for the MCC group at 1 year. Color bar displays effect size.

Activation in prefrontal and parietal cortical regions was observed for the 2-back vs 0-back image contrast in all groups, including MCC participants at baseline and 1 year as well as control participants (Figure 1; eAppendix 1 and eFigure 5 in Supplement 1). There were no significant differences in activation between the groups at baseline (22 HC participants and 40 MCC participants) or between the 2 time points of the MCC group (25 participants), and no associations between cannabis use frequency change and activation at 1 year for the MCC group were significant.

### MID Task Imaging Results

**Figure 2.**
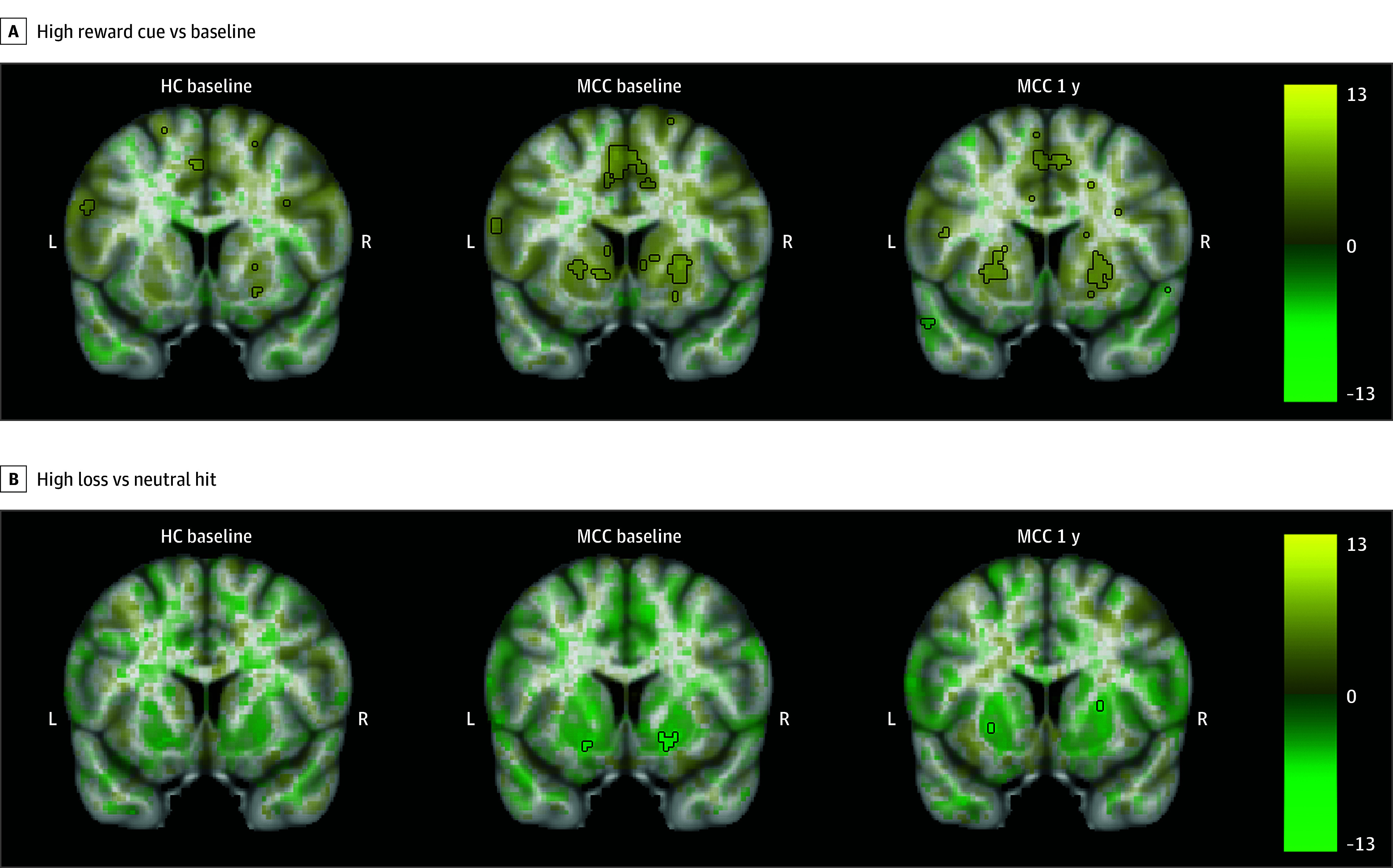
Brain Activation for the Monetary Incentive Delay Task’s High Reward Cue vs Baseline and High Loss vs Neutral Hit Contrast Across Groups and Time Points The healthy control (HC) group at baseline (23 participants), the medical cannabis card (MCC) group at baseline (35 participants), and the MCC group at 1 year (40 participants, except high loss vs neutral hit, with 39 participants) did not show activation differences between the 2 groups at baseline or between the 2 time points of the MCC group. Cannabis use frequency changes were not associated with brain activation at 1 year. Voxel-wise average brain activation, colored by effect size and opacity-scaled by *z* scores with the significance threshold (false-discovery rate *P* < .05) outlined, for the high reward cue vs baseline contrast (A) and the high loss vs neutral hit feedback contrast (B) of the Monetary Incentive Delay task. The *z* thresholds were 3.37 for high reward and undetermined for high loss for the HC group at baseline, 3.24 for high reward and 4.34 for high loss for the MCC group at baseline, and 3.10 for high reward and 4.65 for high loss for the MCC group at 1 year. Note that activation for the other contrasts was below threshold. Color bar displays effect size. An undetermined *z* threshold indicates that no voxel was statistically significant.

MCC participants at baseline and 1 year, as well as controls, showed activation in the bilateral basal ganglia during all cue contrasts, although activation only reached statistical significance during the high reward cue vs baseline contrast (Figure 2; eAppendix 1 and eFigures 6-8 in Supplement 1).^30^ During the reward vs missed reward feedback contrast, activation in the bilateral basal ganglia was observed, while during all other feedback contrasts, deactivation in the bilateral basal ganglia and insula was observed, although only significant in the high loss vs neutral hit contrast. No significant differences in activation were observed between the groups at baseline (23 HC participants and 35 MCC participants) or between the 2 time points of the MCC group (22 participants). Within the MCC group, there were no significant associations between brain activation at 1 year and cannabis use frequency changes.

### SST Task Behavioral and Imaging Results

At baseline, HC participants had significantly faster mean (SD) SSRT (259 [43] milliseconds) compared with MCC participants (276 [40] milliseconds) (β = −16.2; SE, 7.7; *P* = .04), indicating better inhibitory control. MCC participants had a nonsignificant reduction in mean (SD) SSRT from baseline (276 [40] milliseconds) to 1 year (264 [43] milliseconds) (β = −10.0; SE, 5.1; *P* = .05) (eTable 3 in Supplement 1).

**Figure 3.**
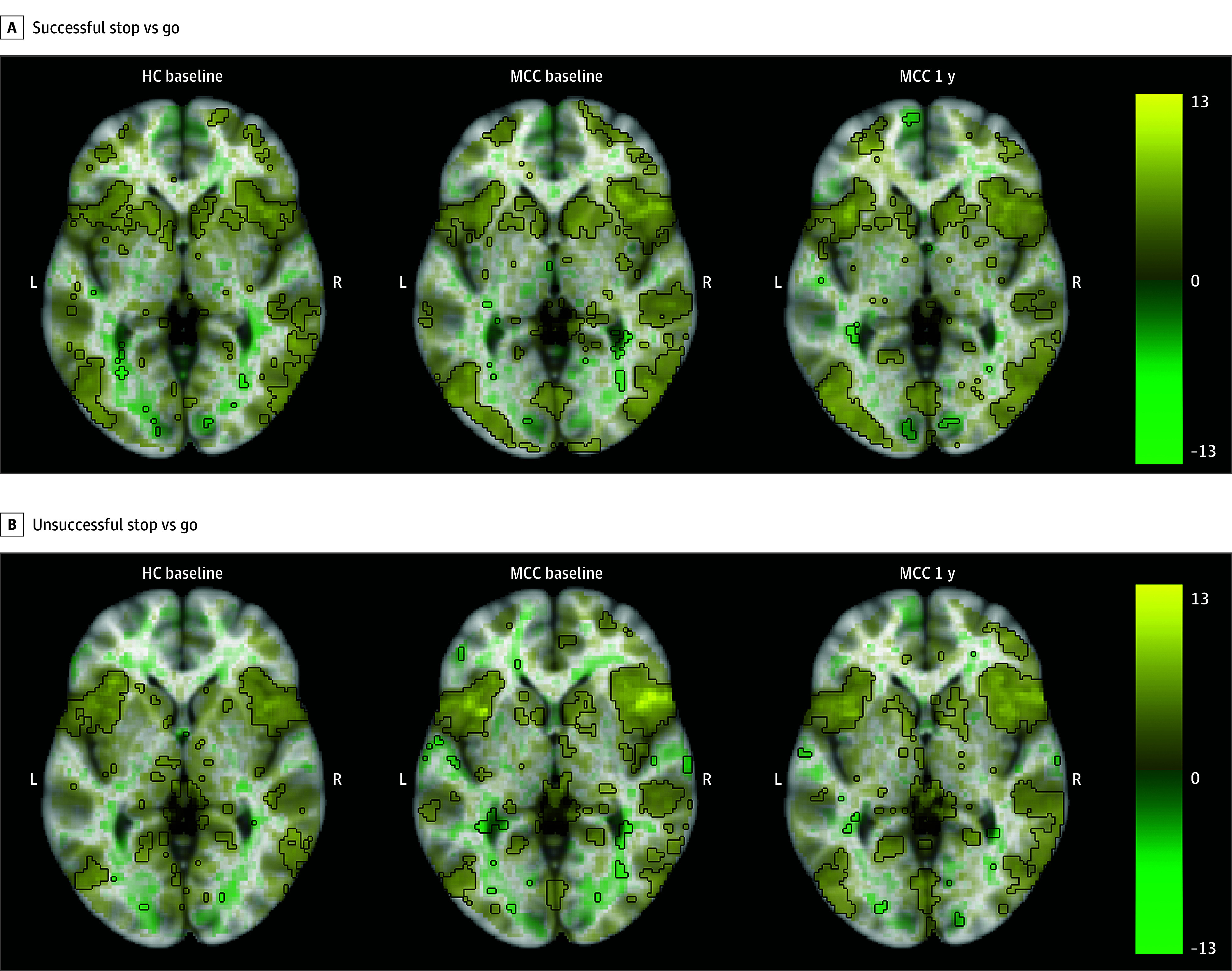
Brain Activation for the Stop Signal Task 2 Stop vs Go Contrasts Across Groups and Time Points The healthy control (HC) group at baseline (25 participants), the medical cannabis card (MCC) group at baseline (40 participants), and the MCC group at 1 year (44 participants) did not show activation differences between the 2 groups at baseline or between the 2 time points of the MCC group. Cannabis use frequency changes were not associated with brain activation at 1 year. Voxel-wise average brain activation, colored by effect size and opacity-scaled by *z* scores with the significance threshold (false-discovery rate *P* < .05) outlined, for the successful stop vs go contrast (A) and the unsuccessful stop vs go contrast (B) for the Stop Signal Task. The *z* thresholds were 2.77 for successful stop vs go and 2.87 for unsuccessful stop vs go for the HC group at baseline, 2.56 for successful stop vs go and 2.62 for unsuccessful stop vs go for the MCC group at baseline, and 2.62 for successful stop vs go and 2.55 for unsuccessful stop vs go for the MCC group at 1 year. Color bar displays effect size.

All groups showed activation in inhibitory control-related regions, including the right inferior frontal gyrus, frontal gyrus, and insula during the correct inhibition, incorrect inhibition, and successful inhibitory control contrasts (Figure 3; eAppendix 1 and eFigures 9-11 in Supplement 1). No differences in activation between the groups at baseline (25 HC participants and 40 MCC participants) or between the 2 time points of the MCC group (26 participants), and no associations of cannabis use frequency change with activation at 1 year for the MCC group were significant.

### Effect of Quality Control on Neuroimaging Results

We note that we removed between 7 and 22 participants based on quality control metrics that were calculated separately for each run. Including additional participants in the analysis by relaxing the framewise displacement threshold from 0.2 to 0.3 or by including all participants regardless of quality control metrics did not significantly change the results (eAppendix 2 and eFigures 12-17 in Supplement 1).

## Discussion

After year-long cannabis use for medical symptoms in adults who newly obtained MCCs, we did not observe functional differences between baseline and brain activation at 1 year during working memory, reward processing, or inhibitory control tasks, nor an association between changes in cannabis use frequency and brain activation at 1 year. Similarly, few significant changes in behavioral performance emerged. This suggests that cannabis use for medical purposes, within the snapshot of cognition captured by these tasks and within a mostly older, White, female, and generally well-educated population, did not have a significant association with brain activation or cognitive performance.

Prior studies have found that cannabis use, especially in adolescents, is associated with impairments in cognitive processes beyond acute intoxication.^11,31,32^ Such studies have largely been cross-sectional, have generally not focused on adults using cannabis for medical purposes, and have focused primarily on heavy cannabis use. Furthermore, conclusions of studies comparing individuals who use cannabis with those who do not often are limited by inherent group differences at the outset. Because participants in this study did not use cannabis heavily at baseline and obtained MCCs for the first time for their medical symptoms, the study was uniquely positioned to examine the brain before and after adults began to use cannabis regularly, a period that is difficult to capture. To our knowledge, this study is among the first to evaluate brain activation differences in an ecologically valid setting in those who began using cannabis for medical symptoms. Brain activation differences were also not found between HC participants and MCC participants at baseline, suggesting that the MCC group did not differ significantly at baseline from those who did not intend to use cannabis.

Memory is one of the most consistently reported processes that is affected by cannabis.^33^ Prior studies comparing those who use cannabis with those who do not have found significant changes in activation of frontal regions during the N-back task,^34,35,36,37^ although it should be noted that other studies did not report statistically significant differences.^38,39^ Reward-related activation has also been implicated in cannabis use, as prior studies have found significant changes in the activation of striatal regions during the MID task.^40,41,42,43,44,45^ Finally, inhibitory control activation differences have been reported,^46,47^ particularly in fronto-basal-ganglia circuits.^48^ However, these studies mainly consist of those who began using cannabis during adolescence or those who use cannabis frequently. Contrary to our initial hypothesis and this literature, our findings indicate that activation to working memory, reward processing, and inhibitory control tasks is largely unchanged in adults using cannabis to alleviate medical symptoms for 1 year. This study population may differ from previous studies of recreational cannabis in participants’ use patterns, motivations for use, age, or other factors.

Overall, the activation patterns of all 3 tasks were consistent with those observed in the literature. The working memory and inhibitory control tasks yielded statistically significant canonical activations in the control and MCC groups at baseline and in the MCC group at 1 year.^20,48,49,50^ The reward processing task also yielded canonical activations for the cue contrasts.^30,50,51,52,53^ Activations to feedback were consistent with previous studies, though the literature is less robust in regards to the feedback contrast.^51,52,53^ Of note, only 2 of the MID contrasts reached statistical significance. This suggests that the response to the task was more heterogeneous in this participant sample, and perhaps a larger sample or a differently designed reward task would have been needed to achieve more robust activation. It was recently noted that the MID task can have low reliability, which is consistent with our findings.^54^

### Limitations

This study should be interpreted in light of its limitations. First, the sample was predominantly female, White, older, and well-educated, which may limit the generalizability of our findings. Future studies should thus focus on recruiting a more diverse sample. It is further possible that the lack of difference in task-based activation was due to limited power. We note that the maximal absolute effect size difference between the 2 time points in the MCC group was smaller than the maximal absolute effect size across the 2 time points for all 3 tasks. Thus, even if we did not detect a difference despite one existing, the change in brain activation after year-long cannabis use would be small. This study took place partly during the COVID-19 pandemic, which caused some participants to opt out of scanning procedures, reducing the sample size of those with neuroimaging data.

Importantly, adult-onset use of cannabis for medical symptoms after obtaining MCCs likely has different neural implications compared with recreational adolescent use. Moreover, comorbid conditions (eg, depression or pain) may influence the impact of cannabis on the brain. While our sample size was too small for a subgroup analysis of each of the symptoms for which participants sought MCCs, future studies should enroll sufficient participants to be able to discover the impact of cannabis within symptoms. Additionally, to emulate the system of medical cannabis in place, participants freely chose cannabis products at local dispensaries. Therefore, it is possible that doses of cannabinoids were too low to observe brain changes. Further research is warranted to understand how differences in product type, amounts, and patterns of use might affect the brain in cannabis users for medical symptoms. Additional limitations are presented in eAppendix 3 in Supplement 1.

## Conclusions

In this cohort study of adults obtaining MCCs for medical symptoms, brain activation during working memory, reward processing, and inhibitory control tasks was not significantly different after year-long cannabis use and no association with changes in cannabis use frequency was noted. Our results suggest that adults who use cannabis, generally with light to moderate use patterns, for symptoms of pain, anxiety, depression, or poor sleep, experience few significant long-term neural associations in these areas of cognition.

## Notes

Supplement 1.
eMethods.
**eFigure 1.** Study Flow Diagram **eFigure 2.** Schematic of the N-back Task **eFigure 3.** Schematic of the MID Task **eFigure 4.** Schematic of the SST Task **eTable 1.** Statistically Significant Difference in Cannabis Use-Related Metrics After 1 Year of Cannabis Use for Medical Symptoms **eTable 2.** No Statistically Significant Difference in N-Back Behavioral Measures Compared With Control or Across Time Except in 2-back Reaction Time Across Time **eTable 3.** Significantly Faster SSRT in Healthy Controls Compared With MCC Participants at Baseline, but no Significant Difference Across Time in MCC Participants **eAppendix 1.** Additional Imaging Results **eFigure 5.** Brain Activation for the N-back Task’s 2-back vs 0-back Contrast Across Groups and Time Points, the Same Contrast as in the Main Article Analyzed in Grayordinate Space **eFigure 6.** Brain Activation for the MID Task’s High Reward Cue vs Baseline Contrast Across Groups and Time Points, the Same Contrast as in the Main Article Analyzed in Grayordinate Space **eFigure 7.** Brain Activation for Additional Contrasts of the MID Task Across Groups and Time Points From the Volumetric Analysis **eFigure 8.** Brain Activation for Additional Contrasts of the MID Task Across Groups and Time Points From the Grayordinate Analysis **eFigure 9.** Brain Activation for the SST Task’s 2 Stop vs Go Contrasts Across Groups and Time Points, the Same Contrasts as in the Main Article Analyzed in Grayordinate Space **eFigure 10.** Brain Activation for an Additional Contrast of the SST Task Across Groups and Time Points From the Volumetric Analysis **eFigure 11.** Brain Activation for an Additional Contrast of the SST Task Across Groups and Time Points From the Grayordinate Analysis **eAppendix 2.** Quality Control and Neuroimaging Results **eFigure 12.** Brain Activation for the N-back Task’s 2-back vs 0-back Contrast Across Groups and Time Points With no Outliers Removed **eFigure 13.** Brain Activation for the N-back Task’s 2-back vs 0-back Contrast Across Groups and Time Points With the FD Cutoff Relaxed to 0.3 **eFigure 14.** Brain Activation for Various Contrasts of the MID Task Across Groups and Time Points With no Outliers Removed **eFigure 15.** Brain Activation for Various Contrasts of the MID Task Across Groups and Time Points With the FD Cutoff Relaxed to 0.3 **eFigure 16.** Brain Activation for the SST Task’s 2 Stop vs Go Contrasts Across Groups and Time Points With no Outliers Removed **eFigure 17.** Brain Activation for the SST Task’s 2 Stop vs Go Contrasts Across Groups and Time Points With the FD Cutoff Relaxed to 0.3 **eAppendix 3.** Supplementary Limitations
eReferences.

Supplement 2.
Data Sharing Statement

## References

[1] Burggren AC, Shirazi A, Ginder N, London ED. Cannabis effects on brain structure, function, and cognition: considerations for medical uses of cannabis and its derivatives. Am J Drug Alcohol Abuse. 2019;45(6):563-579. doi:10.1080/00952990.2019.1634086 31365275 PMC7027431

[2] Batalla A, Bhattacharyya S, Yücel M, . Structural and functional imaging studies in chronic cannabis users: a systematic review of adolescent and adult findings. PLoS One. 2013;8(2):e55821. doi:10.1371/journal.pone.0055821 23390554 PMC3563634

[3] Yanes JA, Riedel MC, Ray KL, . Neuroimaging meta-analysis of cannabis use studies reveals convergent functional alterations in brain regions supporting cognitive control and reward processing. J Psychopharmacol. 2018;32(3):283-295. doi:10.1177/0269881117744995 29338547 PMC5858977

[4] Mason NL, Theunissen EL, Hutten NRPW, . Reduced responsiveness of the reward system is associated with tolerance to cannabis impairment in chronic users. Addict Biol. 2021;26(1):e12870. doi:10.1111/adb.12870 31865628 PMC7757162

[5] Crane NA, Schuster RM, Fusar-Poli P, Gonzalez R. Effects of cannabis on neurocognitive functioning: recent advances, neurodevelopmental influences, and sex differences. Neuropsychol Rev. 2013;23(2):117-137. doi:10.1007/s11065-012-9222-1 23129391 PMC3593817

[6] Lisdahl KM, Wright NE, Kirchner-Medina C, Maple KE, Shollenbarger S. Considering cannabis: the effects of regular cannabis use on neurocognition in adolescents and young adults. Curr Addict Rep. 2014;1(2):144-156. doi:10.1007/s40429-014-0019-6 25013751 PMC4084860

[7] Jernigan TL, Brown SA; ABCD Consortium Coordinators. Introduction. Dev Cogn Neurosci. 2018;32:1-3. doi:10.1016/j.dcn.2018.02.002 29496476 PMC6969247

[8] Mackie K. Distribution of cannabinoid receptors in the central and peripheral nervous system. Handb Exp Pharmacol. 2005;(168):299-325. doi:10.1007/3-540-26573-2_10 16596779

[9] Piomelli D. The molecular logic of endocannabinoid signalling. Nat Rev Neurosci. 2003;4(11):873-884. doi:10.1038/nrn1247 14595399

[10] Martín-Santos R, Fagundo AB, Crippa JA, . Neuroimaging in cannabis use: a systematic review of the literature. Psychol Med. 2010;40(3):383-398. doi:10.1017/S0033291709990729 19627647

[11] Dellazizzo L, Potvin S, Giguère S, Dumais A. Evidence on the acute and residual neurocognitive effects of cannabis use in adolescents and adults: a systematic meta-review of meta-analyses. Addiction. 2022;117(7):1857-1870. doi:10.1111/add.15764 35048456

[12] Volkow ND, Swanson JM, Evins AE, . Effects of cannabis use on human behavior, including cognition, motivation, and psychosis: a review. JAMA Psychiatry. 2016;73(3):292-297. doi:10.1001/jamapsychiatry.2015.3278 26842658

[13] Boehnke KF, Dean O, Haffajee RL, Hosanagar A. U.S. trends in registration for medical cannabis and reasons for use from 2016 to 2020: an observational study. Ann Intern Med. 2022;175(7):945-951. doi:10.7326/M22-0217 35696691 PMC10233658

[14] National Academies of Sciences, Engineering, and Medicine; Health and Medicine Division; Board on Population Health and Public Health Practice; Committee on the Health Effects of Marijuana: An Evidence Review and Research Agenda. Therapeutic Effects of Cannabis and Cannabinoids. National Academies Press; 2017.

[15] Gilman JM, Schuster RM, Potter KW, . Effect of medical marijuana card ownership on pain, insomnia, and affective disorder symptoms in adults: a randomized clinical trial. JAMA Netw Open. 2022;5(3):e222106. doi:10.1001/jamanetworkopen.2022.2106 35302633 PMC8933735

[16] Cooke ME, Potter KW, Jashinski J, . Development of cannabis use disorder in medical cannabis users: a 9-month follow-up of a randomized clinical trial testing effects of medical cannabis card ownership. Front Psychiatry. 2023;14:1083334. doi:10.3389/fpsyt.2023.1083334 36960460 PMC10027723

[17] Filbey FM, Dunlop J, Ketcherside A, . fMRI study of neural sensitization to hedonic stimuli in long-term, daily cannabis users. Hum Brain Mapp. 2016;37(10):3431-3443. doi:10.1002/hbm.23250 27168331 PMC5012952

[18] Schweinsburg AD, Nagel BJ, Schweinsburg BC, Park A, Theilmann RJ, Tapert SF. Abstinent adolescent marijuana users show altered fMRI response during spatial working memory. Psychiatry Res. 2008;163(1):40-51. doi:10.1016/j.pscychresns.2007.04.018 18356027 PMC2832586

[19] Becker B, Wagner D, Gouzoulis-Mayfrank E, Spuentrup E, Daumann J. The impact of early-onset cannabis use on functional brain correlates of working memory. Prog Neuropsychopharmacol Biol Psychiatry. 2010;34(6):837-845. doi:10.1016/j.pnpbp.2010.03.032 20363277

[20] Owen AM, McMillan KM, Laird AR, Bullmore E. N-back working memory paradigm: a meta-analysis of normative functional neuroimaging studies. Hum Brain Mapp. 2005;25(1):46-59. doi:10.1002/hbm.20131 15846822 PMC6871745

[21] Knutson B, Westdorp A, Kaiser E, Hommer D. FMRI visualization of brain activity during a monetary incentive delay task. Neuroimage. 2000;12(1):20-27. doi:10.1006/nimg.2000.0593 10875899

[22] Logan GD, Schachar RJ, Tannock R. Impulsivity and inhibitory control. Psychol Sci. 1997;8(1):60-64. doi:10.1111/j.1467-9280.1997.tb00545.x

[23] Glasser MF, Sotiropoulos SN, Wilson JA, ; WU-Minn HCP Consortium. The minimal preprocessing pipelines for the Human Connectome Project. Neuroimage. 2013;80:105-124. doi:10.1016/j.neuroimage.2013.04.127 23668970 PMC3720813

[24] Esteban O, Markiewicz CJ, Blair RW, . fMRIPrep: a robust preprocessing pipeline for functional MRI. Nat Methods. 2019;16(1):111-116. doi:10.1038/s41592-018-0235-4 30532080 PMC6319393

[25] Friston KJ, Holmes AP, Worsley KJ, Poline JP, Frith CD, Frackowiak RSJ. Statistical parametric maps in functional imaging: a general linear approach. Hum Brain Mapp. 1994;2(4):189-210. doi:10.1002/hbm.460020402

[26] Abraham A, Pedregosa F, Eickenberg M, . Machine learning for neuroimaging with scikit-learn. Front Neuroinform. 2014;8:14. doi:10.3389/fninf.2014.00014 24600388 PMC3930868

[27] Esteban O, Birman D, Schaer M, Koyejo OO, Poldrack RA, Gorgolewski KJ. MRIQC: advancing the automatic prediction of image quality in MRI from unseen sites. PLoS One. 2017;12(9):e0184661. doi:10.1371/journal.pone.0184661 28945803 PMC5612458

[28] Bossier H, Nichols TE, Moerkerke B. Standardized effect sizes and image-based meta-analytical approaches for fMRI data. bioRxiv. Preprint posted online December 6, 2019. doi:10.1101/865881

[29] Hedges LV. Distribution theory for Glass’s estimator of effect size and related estimators. J Educ Behav Stat. 1981;6(2):107-128. doi:10.3102/10769986006002107

[30] Wilson RP, Colizzi M, Bossong MG, Allen P, Kempton M, Bhattacharyya S; MTAC. The neural substrate of reward anticipation in health: a meta-analysis of fMRI findings in the Monetary Incentive Delay Task. Neuropsychol Rev. 2018;28(4):496-506. doi:10.1007/s11065-018-9385-5 30255220 PMC6327084

[31] Gilman JM, Schuster RM, Curran MT, Calderon V, van der Kouwe A, Evins AE. Neural mechanisms of sensitivity to peer information in young adult cannabis users. Cogn Affect Behav Neurosci. 2016;16(4):646-661. doi:10.3758/s13415-016-0421-8 27068178 PMC4955693

[32] Gilman JM, Lee S, Kuster JK, . Variable activation in striatal subregions across components of a social influence task in young adult cannabis users. Brain Behav. 2016;6(5):e00459. doi:10.1002/brb3.459 27257518 PMC4873656

[33] Dougherty DM, Mathias CW, Dawes MA, . Impulsivity, attention, memory, and decision-making among adolescent marijuana users. Psychopharmacology (Berl). 2013;226(2):307-319. doi:10.1007/s00213-012-2908-5 23138434 PMC3581724

[34] Smith AM, Longo CA, Fried PA, Hogan MJ, Cameron I. Effects of marijuana on visuospatial working memory: an fMRI study in young adults. Psychopharmacology (Berl). 2010;210(3):429-438. doi:10.1007/s00213-010-1841-8 20401748

[35] Ma L, Steinberg JL, Bjork JM, . Fronto-striatal effective connectivity of working memory in adults with cannabis use disorder. Psychiatry Res Neuroimaging. 2018;278:21-34. doi:10.1016/j.pscychresns.2018.05.010 29957349 PMC6953485

[36] Taurisano P, Antonucci LA, Fazio L, . Prefrontal activity during working memory is modulated by the interaction of variation in CB1 and COX2 coding genes and correlates with frequency of cannabis use. Cortex. 2016;81:231-238. doi:10.1016/j.cortex.2016.05.010 27261878

[37] Hatchard T, Byron-Alhassan A, Mioduszewski O, . Working overtime: altered functional connectivity in working memory following regular cannabis use in young adults. Int J Ment Health Addict. 2021;19(4):1314-1329. doi:10.1007/s11469-020-00226-y

[38] Cousijn J, Wiers RW, Ridderinkhof KR, van den Brink W, Veltman DJ, Goudriaan AE. Effect of baseline cannabis use and working-memory network function on changes in cannabis use in heavy cannabis users: a prospective fMRI study. Hum Brain Mapp. 2014;35(5):2470-2482. doi:10.1002/hbm.22342 24038570 PMC6869744

[39] Cousijn J, Vingerhoets WAM, Koenders L, . Relationship between working-memory network function and substance use: a 3-year longitudinal fMRI study in heavy cannabis users and controls. Addict Biol. 2014;19(2):282-293. doi:10.1111/adb.12111 24589297

[40] van Hell HH, Vink M, Ossewaarde L, Jager G, Kahn RS, Ramsey NF. Chronic effects of cannabis use on the human reward system: an fMRI study. Eur Neuropsychopharmacol. 2010;20(3):153-163. doi:10.1016/j.euroneuro.2009.11.010 20061126

[41] Spechler PA, Stewart JL, Kuplicki R, Paulus MP; Tulsa 1000 Investigators. Attenuated reward activations associated with cannabis use in anxious/depressed individuals. Transl Psychiatry. 2020;10(1):189. doi:10.1038/s41398-020-0807-9 32541777 PMC7295993

[42] Martz ME, Trucco EM, Cope LM, . Association of marijuana use with blunted nucleus accumbens response to reward anticipation. JAMA Psychiatry. 2016;73(8):838-844. doi:10.1001/jamapsychiatry.2016.1161 27384542 PMC4972653

[43] Enzi B, Lissek S, Edel MA, . Alterations of monetary reward and punishment processing in chronic cannabis users: an FMRI study. PLoS One. 2015;10(3):e0119150. doi:10.1371/journal.pone.0119150 25799565 PMC4370729

[44] Nestor L, Hester R, Garavan H. Increased ventral striatal BOLD activity during non-drug reward anticipation in cannabis users. Neuroimage. 2010;49(1):1133-1143. doi:10.1016/j.neuroimage.2009.07.022 19631753 PMC2764826

[45] Yip SW, DeVito EE, Kober H, Worhunsky PD, Carroll KM, Potenza MN. Pretreatment measures of brain structure and reward-processing brain function in cannabis dependence: an exploratory study of relationships with abstinence during behavioral treatment. Drug Alcohol Depend. 2014;140:33-41. doi:10.1016/j.drugalcdep.2014.03.031 24793365 PMC4057888

[46] Spechler PA, Stewart JL, Kuplicki R, Paulus MP; Tulsa 1000 Investigators. Parsing impulsivity in individuals with anxiety and depression who use cannabis. Drug Alcohol Depend. 2020;217:108289. doi:10.1016/j.drugalcdep.2020.108289 33002704 PMC7736515

[47] Filbey F, Yezhuvath U. Functional connectivity in inhibitory control networks and severity of cannabis use disorder. Am J Drug Alcohol Abuse. 2013;39(6):382-391. doi:10.3109/00952990.2013.841710 24200208 PMC4318502

[48] Verbruggen F, Logan GD. Response inhibition in the stop-signal paradigm. Trends Cogn Sci. 2008;12(11):418-424. doi:10.1016/j.tics.2008.07.005 18799345 PMC2709177

[49] Verbruggen F, Aron AR, Band GP, . A consensus guide to capturing the ability to inhibit actions and impulsive behaviors in the stop-signal task. Elife. 2019;8:e46323. doi:10.7554/eLife.46323 31033438 PMC6533084

[50] Chaarani B, Hahn S, Allgaier N, ; ABCD Consortium. Baseline brain function in the preadolescents of the ABCD Study. Nat Neurosci. 2021;24(8):1176-1186. doi:10.1038/s41593-021-00867-9 34099922 PMC8947197

[51] Oldham S, Murawski C, Fornito A, Youssef G, Yücel M, Lorenzetti V. The anticipation and outcome phases of reward and loss processing: a neuroimaging meta-analysis of the monetary incentive delay task. Hum Brain Mapp. 2018;39(8):3398-3418. doi:10.1002/hbm.24184 29696725 PMC6055646

[52] Knutson B, Fong GW, Adams CM, Varner JL, Hommer D. Dissociation of reward anticipation and outcome with event-related fMRI. Neuroreport. 2001;12(17):3683-3687. doi:10.1097/00001756-200112040-00016 11726774

[53] Demidenko MI, Weigard AS, Ganesan K, . Interactions between methodological and interindividual variability: how Monetary Incentive Delay (MID) task contrast maps vary and impact associations with behavior. Brain Behav. 2021;11(5):e02093. doi:10.1002/brb3.2093 33750042 PMC8119872

[54] Demidenko MI, Mumford JA, Poldrack RA. Impact of analytic decisions on test-retest reliability of individual and group estimates in functional magnetic resonance imaging: a multiverse analysis using the monetary incentive delay task. bioRxiv. Preprint posted online March 20, 2024. doi:10.1101/2024.03.19.585755 PMC12290630 40800476

