# Supplementary material for: Year-Long Cannabis Use for Medical Symptoms and Brain Activation During Cognitive Processes
Source: JAMA Netw Open. 2024 Sep 18;7(9):e2434354. doi: 10.1001/jamanetworkopen.2024.34354 (PMC11411392; doi:10.1001/jamanetworkopen.2024.34354)
Supplement: Supplement 2. — Data Sharing Statement [file jamanetwopen-e2434354-s002.pdf]

Data Sharing Statement

Burdinski. Year-Long Cannabis Use for Medical Symptoms and Brain Activation During Cognitive Processes. *JAMA Netw Open*. Published September 18, 2024.
doi:10.1001/jamanetworkopen.2024.34354

Data
Data available: Yes
Data types: Deidentified participant data
How to access data: The data are shared with and accessible through the ENIGMA consortium: https://enigma.ini.usc.edu/
When available: With publication

Supporting Documents
Document types: Statistical/analytic code
How to access documents: All preprocessing and analysis code is available here: https://github.com/burdinskid13/cannabis-paper.
When available: With publication

Additional Information
Who can access the data: The data will be available to those researchers whose proposed use of the data has been approved by the ENIGMA consortium.
Types of analyses: The data will be made available for analyses in alignment with guidelines from the ENIGMA consortium.
Mechanisms of data availability: The data will be made available with support of the ENIGMA consortium.
